# P-2362. Risk Factors for Persistent and Recurrent CMV DNAemia in a Real-World Cohort of SOT and HSCT Patients Receiving Maribavir

**DOI:** 10.1093/ofid/ofae631.2513

**Published:** 2025-01-29

**Authors:** Chelsea Morinishi, Joanna M Schaenman, Omer E Beaird, Pryce Gaynor, Ashrit Multani, Christine Pham, Lauren Yanagimoto-Ogawa

**Affiliations:** David Geffen School of Medicine at UCLA, Los Angeles, California; University of California Los Angeles, David Geffen School of Medicine, Los Angeles, California; UCLA, Los Angeles, California; UCLA, Los Angeles, California; David Geffen School of Medicine at UCLA, Los Angeles, California; University of California, Los Angeles; David School of Medicine/University of California, Los Angeles, Los Angeles, California; David Geffen School of Medicine at UCLA, Los Angeles, California

## Abstract

**Background:**

Refractory/resistant (R/R) CMV infection, defined by persistent symptoms or DNAemia after at least two weeks of antiviral therapy, is associated with high rates of rejection and mortality in transplant patients. Maribavir (MBV) is a newer agent with demonstrated efficacy in achieving DNAemia clearance without the bone marrow suppression associated with conventional first line therapy. However, there is less data on the risk factors for treatment failure with MBV. We sought to evaluate clinical outcomes of MBV in transplant patients in a real-world patient cohort.

**Table 1:**
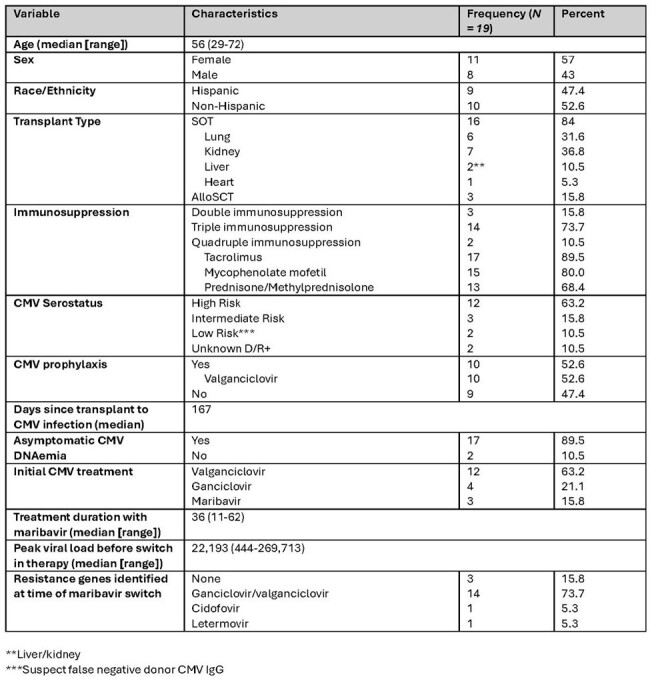
Demographic Characteristics

**Methods:**

A retrospective chart review was conducted of adult solid organ transplant (SOT) and hematopoietic stem cell transplant (HSCT) recipients who received MBV for R/R CMV infection at a high-volume transplant center from 2021-2023. The main treatment outcome was failure (defined as persistence of DNAemia requiring a switch in therapy).

**Table 2:**
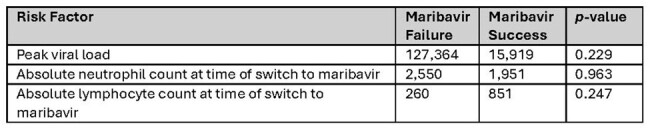
Risk Factors for Maribavir Failure

**Results:**

Nineteen patients received MBV for CMV DNAemia or tissue-invasive disease. Overall, 84% were SOT, 63% had high-risk CMV serostatus, and 90% had asymptomatic DNAemia. Twenty-six percent of patients experienced MBV failure. The median absolute lymphocyte count (ALC) was 852 µL at MBV switch in those with successful treatment compared with 262 µL in those with MBV failure, although this did not reach statistical significance. Peak viral load was also not significantly associated with MBV failure. Five patients later developed MBV resistance by commercial genotype sequencing.

**Conclusion:**

Real world use of MBV demonstrated comparable rates of viral clearance with clinical trial data in SOT and HSCT patients. While not statistically significant, there was a trend towards MBV failure in patients with lower ALC. Future studies may be useful to determine if switching to MBV before onset of worsening lymphopenia related to first line therapy would impact the rate of treatment failure and MBV resistance. The lack of association between VL and virologic response also suggests that MBV may be safely used in patients regardless of viral load.

**Disclosures:**

Joanna M. Schaenman, MD, PhD, FAST, Eurofins Viracor: Honoraria|F2G: Grant/Research Support|MedCure: Advisor/Consultant|Moderna: Clinical trial support to institution|OneLegacy: Advisor/Consultant

